# How to Safely Deliver Stereotactic Ablative Radiotherapy With High-Dose or Multi-agent Systemic Therapy

**DOI:** 10.7759/cureus.97847

**Published:** 2025-11-26

**Authors:** Mark E Bernard

**Affiliations:** 1 Radiation Oncology, University of Kentucky, Lexington, USA

**Keywords:** concurrent, oilgoprogressive, oligometastatic, sbrt, systemic therapy

## Abstract

Background: While systemic therapy remains the backbone treatment for patients with stage IV cancer, local ablative treatments are often given to improve survival. Common local ablative treatments consist of stereotactic ablative radiotherapy (SABR), surgical metastasectomy, or interventional ablative techniques. SABR has the advantage of being able to give treatment without pausing systemic therapy.

Aims and objectives: This study serves to evaluate the efficacy of SABR given concurrently with high-dose and multi-agent systemic therapy during the off week of systemic therapy.

Methods: A retrospective review of patients between 2023 and 2025 treated with systemic therapy with concurrent SABR during the off week of systemic therapy was conducted. Radiation-induced side effects were analyzed using Common Terminology Criteria for Adverse Events (CTCAE) version 5.0.

Results: Six patients with 12 lesions were included in this study. The five different systemic therapy agents used were 1. carboplatin and paclitaxel and pembrolizumab (one patient), 2. fluorouracil & panitumumab (one patient), 3. capecitabine (one patient), 4. FOLFIRI (folinic acid, fluorouracil, and irinotecan) (two patients), and 5. 5-FOLFIRINOX (folinic acid, fluorouracil, irinotecan, and oxaliplatin) (one patient). Four patients were oligometastatic, and two were oligoprogressive. The most common SABR target was the lung, and the most common SABR regimen was 45 Gy in three fractions. Median planning target volume (PTV) was 10cc. The median time from the last systemic therapy infusion to SBRT was seven days. The median time from the last SBRT fraction to the next systemic therapy infusion was one day. Median follow-up was 7.3 months. There were no grade three or higher toxicities reported. One patient had grade one radiation side effects consisting of pain. One patient had diarrhea described as being mild and considered to be grade one. Out of the twelve lesions treated, there were three distant failures and one local/regional failure. Median overall survival was 18.9 months.

Conclusion: SABR given concurrently with multi-agent and high-dose systemic therapy during the off week of therapy is a safe and efficacious option. This treatment technique allows patients to remain on systemic therapy without any interruptions. Therefore, SABR can be a preferred local ablative treatment for patients with oligometastatic and oligoprogressive cancer while on concurrent systemic therapy.

## Introduction

The standard of care treatment for most stage IV solid tumors is systemic therapy, which can consist of chemotherapy, immune checkpoint inhibitors, targeted therapy, or any combination of these [1-2]. Metastasectomy options are often given to improve outcomes for patients who are oligometastatic or have oligoprogression [3-5]. Oligometastatic is defined as patients with one to five sites of metastatic involvement [4]. Oligoprogression is defined as one to five sites of progression, irrespective of the total number of metastatic sites [5]. Stereotactic ablative radiotherapy (SABR), surgical metastasectomy, or interventional ablative techniques are local options to improve outcomes for oligometastatic or oligoprogressive patients [3-4, 6-7]. However, patients who receive surgical metastasectomy or interventional ablations may have to be off systemic therapy to prevent complications from the local treatment. This may also be true for interventional ablative techniques. SABR has the advantage of allowing the patient to continue systemic therapy without systemic therapy interruptions [8].

The timing of SABR, with concurrent systemic therapy, can determine its safety or efficacy. There are several systemic therapy options that are also radiation sensitizers. Therefore, a radiation oncologist may be concerned about causing morbidity to the normal tissue. However, given the half-lives of systemic drugs, there is an optimal time window where the levels are near their nadir and thus should not significantly increase morbidity. Additionally, if the SABR is given closely to the next administration of systemic therapy, this may increase the chances of synergistic tumor cell kill. Therefore, the hypothesis is that SABR can be safely given during the off week of systemic therapy. This would only apply to patients who had at least two weeks between systemic therapy infusions. For example, if a patient is on an every 14-day infusion, the off week in which SABR would be given would be on or during days 8 to 14. If a patient is on every 21-day infusion, the off week in which SABR would be given would be on days 8 to 14 or days 15 to 21. This publication serves as an illustration of how to deliver SABR safely with high-dose or multi-agent systemic therapy.

## Materials and methods

This study was conducted with the approval of the University of Kentucky Office's institutional review board (study approval number 107231). A retrospective review of patients treated between 2023 and 2025 with SABR given concurrently with high-dose or multi-agent systemic therapy was conducted. The SABR was given during the "off week" of systemic therapy. The off-week was defined as the seven-day period when the patient did not receive systemic therapy and would only work for every 14-day or higher schedule. SABR was defined as treatment consisting of 6 Gy or more per treatment for a total of one to five treatments. SABR was performed on our TrueBeam® (Varian Medical Systems, Palo Alto, CA, USA) linear accelerator only. Patient variables collected were age, primary histology, oligometastatic vs. oligoprogressive cancer status, systemic therapy treatment-related variables, radiotherapy-related variables, and acute radiation-related toxicity. Overall survival was defined as the time between the date of diagnosis and the date of death or last follow-up. Follow-up was defined as the time between the date of the last SABR treatment and the last follow-up or death. Local control was defined as recurrence within the planning target volume (PTV). Acute toxicity was defined using the Common Terminology Criteria for Adverse Events (CTCAE) version 5.0 [9].

## Results

A total of six patients with 12 metastases treated with SABR were identified. Their demographics are seen in Table 1 below.

**Table 1:** Patient demographics NSCLC: non-small cell lung cancer

Variable	
Median age (in years, during treatment)	67 (41–70)
Primary histology	Breast cancer (1 patient)
	Descending colon cancer (1 patient)
	Melanoma (1 patient)
	NSCLC (1 patient)
	Rectal cancer (2 patients)
Oligometastatic status	Oligometastatic (4 patients)
	Oligoprogressive (2 patients)

Patients in this analysis were treated with a variety of systemic therapy agents, but most of them were fluorouracil-based. The most common frequency was every 14-day dosing. SABR was delivered to several different sites, but the most common organ was the lung. The most common SABR regimen was 45 Gy in three fractions done every other day. Median PTV was 10 cc (range: 5.7 - 66cc). The median interval from last systemic therapy to first SABR treatment was seven days (range: six to 11 days), and the median interval from last SABR treatment to next systemic therapy was one day (range: one to five days).

Case 1 was a 68-year-old male patient who was diagnosed with oligometastatic non-small cell lung cancer (squamous cell carcinoma). He started carboplatin and paclitaxel for one cycle while inpatient and eventually had pembrolizumab added on his second cycle as an outpatient, given every 21 days. During cycle three and before cycle four, he simultaneously received 50 Gy in five daily fractions to the left upper lung and right upper lung and 30 Gy in five daily fractions to a mediastinal lymph node. He had no acute side effects related to his radiation treatment, and at a follow-up of 6.4 months, he had no local or distant failure.

Case 2 was a 68-year-old female patient with oligometastatic rectal cancer with a retroperitoneal lymph node recurrence. She was on fluorouracil and panitumumab every fourteen days. She received SABR consisting of 35 Gy to the PTV and 40 Gy to the gross tumor volume (GTV), done simultaneously, in five fractions every other day. Her medical oncologist delayed her next systemic therapy by one week, and thus the next cycle began five days after her last SABR fraction. She had no acute side effects related to SABR. For this analysis, at the last follow-up of 19.1 months, she had no local recurrence, but during this time she had a distant recurrence in the left lung treated with SABR.

Case 3 was a 65-year-old female patient with oligoprogressive breast cancer in the liver who was receiving capecitabine for seven days, and then she was off for seven days. She received SABR to a liver metastasis consisting of 45 Gy in three every-other-day fractions. She did have mild diarrhea associated with SABR and considered to be grade 1. At a follow-up of 12.4 months, she developed a distant recurrence.

Case 4 was a 41-year-old male patient with oligoprogressive descending colon cancer who was on every 14-day dosing of FOLIRI. During his FOLFIRI (folinic acid, fluorouracil, and irinotecan), he had two right upper lung metastases that simultaneously received 45 Gy in three fractions every other day. He developed both local/regional and distant recurrence and subsequently expired. He did not have any acute side effects associated with his SABR.

Case 5 was a unique case because this patient had biopsy-proven melanoma lung metastasis but was receiving FOLFIRI and bevacizumab for metastatic colon cancer. This patient was allowed to receive SABR for oligometastatic melanoma while remaining on systemic therapy for his second primary; the bevacizumab was held before SABR but eventually resumed after SABR. At a follow-up of 8.2 months, he had no local or distant recurrences and no acute side effects related to SABR.

Case 6 was a 46-year-old male patient with oligometastatic rectal cancer who was on FOLFIRINOX (folinic acid, fluorouracil, irinotecan, and oxaliplatin) and received SABR to a right upper lung metastasis. He developed grade one pain described as being a sharp chest pain with deep breaths. At a limited follow-up of one month, he had no local or distant recurrences. Table 2 lists the treatment-related factors in more detail.

**Table 2:** Treatment-related variables SABR: stereotactic ablative radiotherapy; S_SABR: time interval from systemic therapy administration to first SABR treatment; SABR_S: time interval from last SABR to next systemic therapy administration; FOLFIRI: folinic acid, fluorouracil, and irinotecan; FOLFIRINOX: folinic acid, fluorouracil, irinotecan, and oxaliplatin; NSCLC: non-small cell lung cancer; Q: every; QD: every day; QOD: every other day; RP: retroperitoneal

Case	Primary	Systemic Therapy	Frequency (Days)	SABR Location	SABR Dose (Gy)	SBRT Fx	Frequency	S_SABR (Days)	SABR_S (Days)
1	NSCLC	Carboplatin paclitaxel pembrolizumab	Q21	Left upper lung	50	5	QD	11	4
				Right upper lung	50	5	QD	11	4
				Mediastinal lymph node	30	5	QD	11	4
2	Rectal	Fluorouracil & panitumumab	Q14	Left RP lymph node	35/40	5	QOD	6	5
3	Breast	Capecitabine	Q14 (7 Days on 7 days off)	Liver	45	3	QOD	1	2
4	Descending colon	FOLFIRI	Q14	Right upper lung	45	3	QOD	7	1
				Right lower lung	45	3	QOD	7	1
5	Melanoma	FOLFIRI	Q14	Right upper lung	45	3	QOD	7	1
				Right middle lung	30	3	QOD	7	1
				Right lower lung	45	3	QOD	7	1
				Left upper lung	45	3	QOD	7	1
6	Rectum	FOLFIRINOX	Q14	Right upper lung	45	3	QOD	8	1

At a median follow-up of 7.3 months (range: 1.0-19.1 months), the median overall survival was 18.9 months (range: 3.5-166.2 months). Only one patient had a local/regional failure, and three patients had distant failure. There were no acute grade three or higher CTCAE radiation-related side effects [9]. One patient had grade one radiation side effects consisting of pain. One patient had diarrhea described as being mild and considered to be grade one.

## Discussion

This single-institution experience offers an option on how to deliver SABR for patients who are receiving concurrent high-dose or multi-agent systemic therapy. Oligometastatic and oligoprogressive stage IV cancer patients can benefit from local therapy options, which include surgical metastasectomy, interventional ablative techniques, or SABR [3-4, 6-7]. Surgical metastasectomy often requires temporary discontinuation of systemic therapy four weeks prior to surgical intervention and several weeks after surgical intervention to allow for healing. Due to this potential long delay, this option could possibly lead to the progression of microscopic metastasis. Interventional ablative techniques may also require withholding of systemic therapy. SABR provides a great option to ablate targeted metastasis and has been shown to improve both overall survival and disease-free survival for patients with oligometastatic cancer. While SABR uses very high doses per treatment, systemic therapy does not need to be held, which gives this non-invasive treatment an advantage over other local ablative treatments. However, the timing of SABR is key to preventing radiation-induced morbidity.

This article highlights that the best time to give SABR for patients who are on multi-agent or high-dose systemic therapy is during the “off week” of treatment. Meaning, if a patient is receiving systemic therapy every 14 days, the best time to start and complete three to five fraction SABR would be on days eight through 13. There was no grade three-related radiation-induced morbidity, and only two, possibly three, patients had grade one morbidity. These low rates of side effects are encouraging, especially since the median time from last systemic to first SABR treatment was seven days and the median time from last SABR to next systemic therapy was two days. The low rates of radiation-induced morbidity are likely due to the SABR being delivered during the off week of systemic therapy, which is during the nadir, or lower concentration of the systemic therapy. Below in Table 3 are the different systemic therapy agents used and their respective approximate half-lives.

**Table 3:** Half-life values for various systemic therapy agents

Systemic agent	Half life
Capecitabine	~ 0.75 hour
Carboplatin	2.6 to 5.9 hours
Fluorouracil	8 to 20 minutes
Irinotecan	6 to 12 hours
Oxaliplatin	Alpha phase: 0.43 hours
	Beta phase: 16.8 hours
	Terminal: 392 hours
Paclitaxel	3-hour infusion: ~13 to 20 hours
	24-hour infusion: ~16 to 53 hours
Panitumumab	~7.5 days (range: ~4 to ~11 days)
Pembrolizumab	22 days

Since the median time from last systemic to first SABR treatment was seven days, by the time the first SABR treatment was given, the systemic therapy concentration was very close to the nadir or had decreased significantly. This is why it is believed the SABR treatment had no grade three or higher side effects. Interestingly, the absence of grade three or higher side effects was shown despite the median time from last SABR to next systemic therapy being one day. This may be related to the SABR’s ability to conform the high doses of radiation to the target while sparing the adjacent critical organs.

Kunos et al. conducted a prospective dose escalation phase I evaluating SABR with concurrent gemcitabine and carboplatin for patients with persistent or recurrent gynecologic cancer [7]. Carboplatin area under the curve (AUC) of 4 and gemcitabine 600 mg/m² were the maximum tolerated doses preceding SABR. In terms of acute grade three toxicity, there were four metabolic toxicity events (hyperkalemia and hypokalemia), four hematologic events, and one episode of anaphylaxis. In terms of acute grade four toxicity events, there was one neutropenic and one hypokalemic event. These toxicity events were most likely related to their systemic therapy and not the SABR, since SABR volumes are relatively small and should not create a significant effect upon metabolic or hematologic systems. However, this study did report that one patient had a late grade three rectovaginal fistula, which occurred 16 months after trial therapy, and this was likely attributable more so to SABR. The small series now being reported did not show any acute grade 3 or higher CTCAE morbidity. These differences may be due to the following reasons. First, Kunos et al. gave SABR the day after receiving gemcitabine and carboplatin, and SABR was done daily. Patients in this retrospective cohort received their first SABR treatment at a median of seven days (range: six to 11 days) from their systemic therapy, and all but one received every-other-day treatment. The longer time from systemic to first SABR in this study could be a reason the grade three morbidity rate was lower than Kunos et al. The every-other-day SABR frequency in this study, rather than the daily one used in the study by Kunos et al., could also have allowed for better normal tissue repair, resulting in lower morbidity rates. Lastly, Kunos et al.'s study was a prospective trial that captures evaluable endpoints better than retrospective series. Interestingly, the patients in this retrospective series had a low acute grade three or higher toxicity rate despite receiving a much higher radiation dose when compared to Kunos et al.

Georgetown University conducted a pilot trial showing the safety of SABR with concurrent full-dose gemcitabine [8]. Eleven patients received 1000 mg/m² gemcitabine for six cycles. Each cycle consisted of weekly gemcitabine for three weeks (Cycle 1, days 1, 8, & 15), and then an off-week (Cycle 1, days 22 - 27) without chemotherapy given. SABR, consisting of 25 Gy in five daily treatments, was given during this off-week (Cycle 1, days 22 - 27). There were no grade three or greater morbidity events attributable to SABR, nor were there delays for Cycle 2 of gemcitabine. They included an endoscopic evaluation to look for mucosal morbidity, and there were no grade two or higher mucosal toxicities. Their treatment strategy of delivering SABR during the off week is similar to the treatment strategy delivered for patients in this study. Also, their acute toxicity results match the results of this retrospective series. However, the SABR schemes in this retrospective series delivered a much higher dose and yet had low rates of morbidity.

This retrospective study does have limitations. Only seven patients with 13 lesions were evaluated, and thus, the applicability of this treatment scheme needs to be evaluated with the physician's discretion. Also, different systemic therapy agents were used. Lastly, retrospective assessment of treatment-related morbidity is not as robust as prospective assessment of treatment-related toxicity. Thus, the small number of patients combined with multiple systemic therapy agents makes broad applicability difficult.

## Conclusions

SABR can be safely given with concurrent multi-agent and high-dose systemic therapy if the SABR is given on and during the off week of systemic therapy. This requires patients to be on an every 14-day or more dosing schedule. However, if they are on an every seven-day schedule, a single fraction may be feasible. This retrospective series also shows the advantages SABR has over interventional ablative techniques or surgical metastasectomy. This retrospective series supports our upcoming prospective trial evaluating SABR given concurrently with immune checkpoint inhibitor-based therapy, many of whom may receive both an immune checkpoint inhibitor and chemotherapy concurrently with SABR. This trial should provide prospective evidence on the use of SABR given concurrently with systemic therapy. As systemic therapy continues to improve, SABR can continue to be thought of as a first-line treatment option for local therapy for oligometastatic or oligoprogressive patients.
